# Role of *sgcR3 *in positive regulation of enediyne antibiotic C-1027 production of *Streptomyces globisporus *C-1027

**DOI:** 10.1186/1471-2180-9-14

**Published:** 2009-01-22

**Authors:** Lifei Wang, Yunfeng Hu, Yanjuan Zhang, Songmei Wang, Zhihui Cui, Yi Bao, Wei Jiang, Bin Hong

**Affiliations:** 1 Institute of Medicinal Biotechnology, Chinese Academy of Medical Sciences & Peking Union Medical College, Beijing 100050, PR China

## Abstract

**Background:**

C-1027, produced by *Streptomyces globisporus *C-1027, is one of the most potent antitumoral agents. The biosynthetic gene cluster of C-1027, previously cloned and sequenced, contains at least three putative regulatory genes, i.e. *sgcR1*, *sgcR2 *and *sgcR3*. The predicted gene products of these genes share sequence similarities to StrR, regulators of AraC/XylS family and TylR. The purpose of this study was to investigate the role of *sgcR3 *in C-1027 biosynthesis.

**Results:**

Overexpression of *sgcR3 *in *S. globisporus *C-1027 resulted in a 30–40% increase in C-1027 production. Consistent with this, disruption of *sgcR3 *abolished C-1027 production. Complementation of the *sgcR3*-disrupted strain R3KO with intact *sgcR3 *gene could restore C-1027 production. The results from real time RT-PCR analysis in R3KO mutant and wild type strain indicated that not only transcripts of biosynthetic structural genes such as *sgcA1 *and *sgcC4*, but also putative regulatory genes, *sgcR1 *and *sgcR2*, were significantly decreased in R3KO mutant. The cross-complementation studies showed that *sgcR1R2 *could functionally complement *sgcR3 *disruption *in trans*. Purified N-terminal His_10_-tagged SgcR3 showed specific DNA-binding activity to the promoter region of *sgcR1R2*.

**Conclusion:**

The role of SgcR3 has been proved to be a positive regulator of C-1027 biosynthesis in *S. globisporus *C-1027. SgcR3 occupies a higher level than SgcR1 and SgcR2 in the regulatory hierarchy that controls C-1027 production and activates the transcription of *sgcR1 *and *sgcR2 *by binding directly to the promoter region of *sgcR1R2*.

## Background

C-1027, also called lidamycin, is a chromoprotein antitumor antibiotic produced by *Streptomyces globisporus *C-1027 [1]. As a member of the enediyne family characterized by two acetylenic groups conjugated to a double bond within a 9- or 10-membered ring, C-1027 is 1,000 times more potent than adriamycin, one of the most effective chemotherapeutic agents [2]. C-1027 is a complex consisting of a 1:1 non-covalently associated mixture of an apoprotein and a 9-membered enediyne chromophore. The chromophore of the enediyne family can undergo a rearrangement to form a transient benzenoid diradical species that can abstract hydrogen atoms from DNA to initiate a cascade leading to DNA breaks, ultimately leading to cell death [3,4]. This novel mode of action has attracted great interest in developing these compounds into therapeutic agents for cancer. A CD33 monoclonal antibody (mAB)-calicheamicin (CAL) conjugate (Mylotarg) and neocarzinostatin (NCS) conjugated with poly (styrene-co-maleic acid) (SMANCS) were approved in the USA [5] and in Japan [6], respectively. Recently, C-1027 has entered phase II clinical trial in China [7]. Appreciation of the immense pharmacological potential of enediynes has led to a demand for the economical production of C-1027 and its analogues at an industrial scale.

Control of secondary metabolite production in streptomycetes and related actinomycetes is a complex process involving multiple levels of regulation in response to environmental factors [For review, see [8,9]]. In most cases that have been studied in detail, the final checkpoint in production of a secondary metabolite is a pathway-specific transcriptional regulatory gene situated in the biosynthetic cluster. Remarkable progress has been made in dissecting the functions of the pathway-specific regulators. For example, ActII-ORF4 regulates transcription from the actinorhodin biosynthetic genes of *S. coelicolor *[10,11] and StrR controls the streptomycin biosynthetic cluster of *S. griseus *[12,13]. Recently, along with the tremendous increase in sequence information for secondary metabolic gene clusters, more and more clusters with multiple cluster-situated regulators were reported (e.g., [14-17]). The best studied multiple pathway-specific regulatory cascade involves remarkably five regulatory genes in tylosin biosynthetic gene cluster of *S. fradiae*, and a model for their regulation has been proposed [14,18-23]. Deciphering the complexity of these pathway-specific regulatory networks is of great interest not only for better understanding of the antibiotic regulatory mechanism, but also for providing new strategy for targeted genetic engineering of antibiotic producing strains.

**Figure 1:**
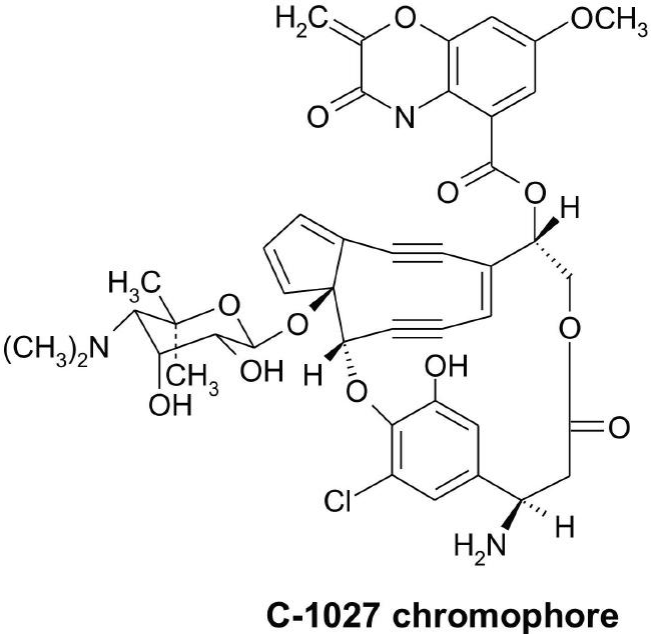
**Structure of C-1027 chromophore**.

**Figure 2:**
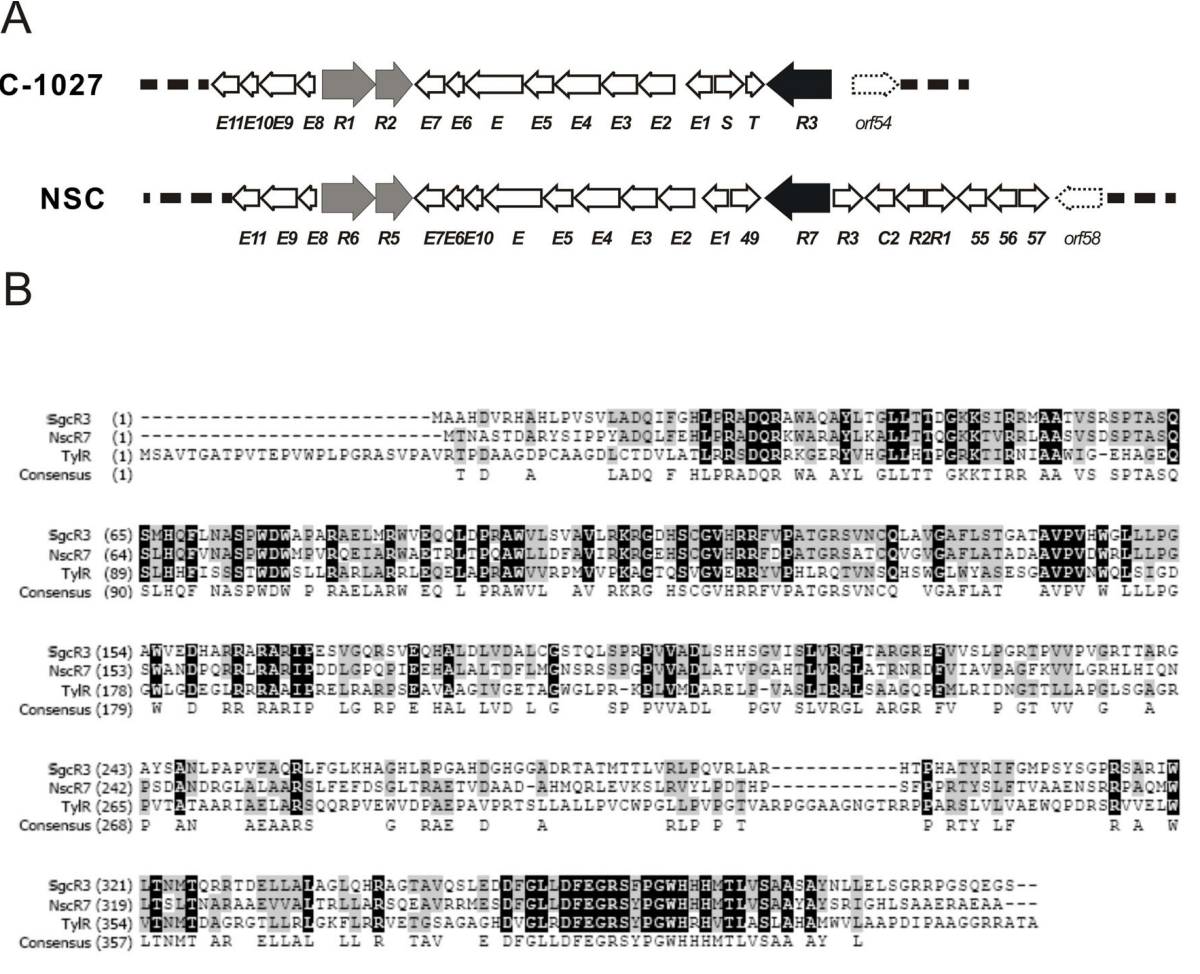
**Comparison of two 9-membered enediyne (C-1027 and NCS) biosynthetic gene clusters around the genes of enediyne PKS (*sgcE *and *ncsE*) (A) and amino acid sequence alignment for SgcR3 (B)**. A, Open reading frames are indicated by arrows. Homologue genes of regulatory *sgcR1*, *sgcR2 *and *sgcR3 *identified by sequence analysis are shown in grey or black. Genes outside of the clusters are marked by broken line arrows. B, The multialignment of *S*. *globisporus *C-1027 SgcR3, *S*. *carzinostaticus *ATCC 15944 NscR7 and *S. fradiae *TylR. Identical residues are highlighted in black and similar residues are shaded. The numbers indicate amino acid positions.

C-1027 nonpeptidic chromophore is a structure of an enediyne core, a deoxy aminosugar, a β-amino acid and a benzoxazolinate (Fig. 1) [7]. The biosynthetic gene cluster for C-1027, which is the first cloned enediyne gene cluster, contains a total of 56 open reading frames (ORFs) in a region of 75 kbp [24,25]. Bioinformatic analysis and biochemical studies revealed a distinct iterative type I enediyne polyketide synthase (SgcE) and provided a convergent biosynthetic strategy for C-1027 from four biosynthetic building blocks [25]. Further cloning and characterization of biosynthetic gene clusters for four other enediynes (CAL [26], NCS [27], maduropeptin (MDP) [28] and dynemicin [29]) confirmed the unifying paradigm for enediyne biogenesis. In accordance with the complexity of the biosynthetic process, there are no fewer than three ORFs annotated as transcriptional regulators in each known enediyne antibiotic biosynthetic cluster. At least three putative regulatory genes (*sgcR1*, *sgcR2 *and *sgcR3*) associated with the C-1027 biosynthetic gene cluster of *S. globisporus *C-1027 were annotated in the earlier work by sequence analysis [25]. Furthermore, the biosynthetic gene clusters for two 9-membered enediynes produced by streptomycetes (C-1027 and NCS) show high similarity in the organization of genes around these regulatory genes (Fig. 2A). Despite chromophore structural uniqueness, all homologues of three genes are located adjacent to the genes of enediyne PKSs (*sgcE *and *ncsE*) and the tailoring enzymes (E1 to E11), which are responsible for the biosynthesis of enediyne core. However, almost no cognitional knowledge was acquired about the transcriptional regulation of enediyne antibiotic production prior to the present work.

Hereby we investigated the role of *sgcR3 *in C-1027 biosynthesis, and provided an initial understanding of pathway-specific regulatory network of *sgcR1*, *sgcR2 *and *sgcR3 *in *S. globisporus *C-1027.

## Results

### Overexpression of *sgcR3 *increased the production of C-1027

**Figure 3:**
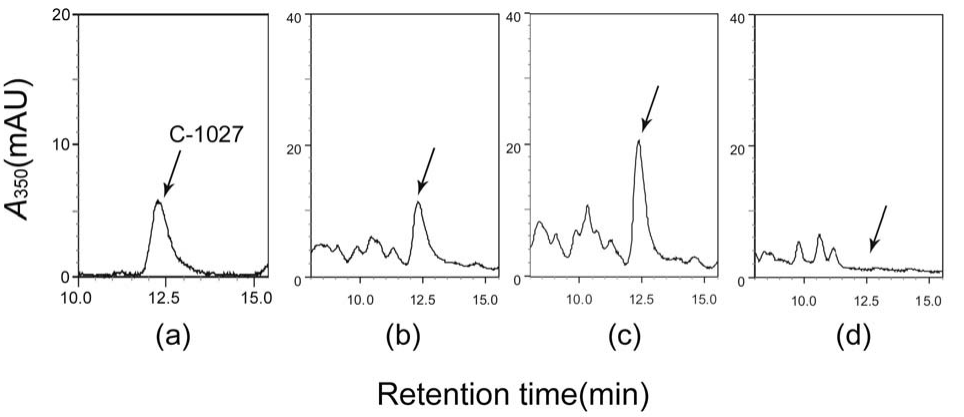
**Determination of C-1027 production in *sgcR3 *overexpression strain and disruption strain R3KO**. HPLC analysis of C-1027 chromophore standard (a), C-1027 produced by wild type strain (b), one of *sgcR3 *overexpression strains (c) and R3KO mutant (d) are shown.

Computer-assisted analysis of the *sgcR3 *gene product (395 aa) showed a high sequence similarity (33% identities and 47% positives) within the whole length of protein TylR of *S. fradiae *(Fig. 2B), a pathway-specific global activator of *tyl *cluster [20,23]. To investigate the function of *sgcR3*, the expression plasmid of *sgcR3 *associated with its native promoter, named pKCR3 (see Methods), was constructed based on the multi-copy pKC1139 [30] and then introduced into *S. globisporus *C-1027 by conjugation. Thereafter, the resultant *sgcR3 *overexpression strains were fermented by incubation in liquid medium FMC-1027-1 (see Methods). The antibacterial bioassay against *Bacillus subtilis *CMCC(B) 63501 (data not shown) and the HPLC analysis indicated that the pKCR3 led to a 30–40% increase in C-1027 production (Fig. 3c) in comparison to that in wild type strain (Fig. 3b), whereas C-1027 production level detected in the wild type strain with the parental vector pKC1139 had no difference. Therefore, the result suggested that the function of *sgcR3 *could be positive for C-1027 biosynthesis in *S. globisporus *C-1027.

### Inactivation and complementation of *sgcR3*

**Figure 4:**
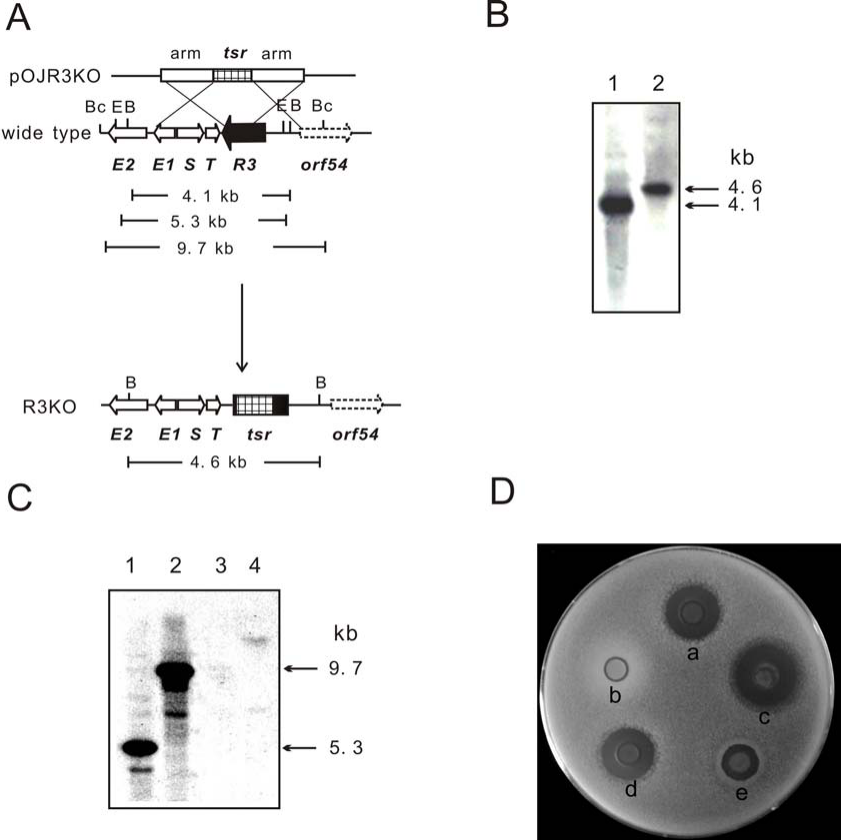
**Inactivation and complementation of *sgcR3***. A, The plasmid pOJR3KO, constructed for *sgcR3 *inactivation as described in Methods, was used for gene disruption. Predicted restriction enzyme polymorphism caused by gene replacement is shown. B, *Bam*HI; Bc, *Bcl*I; E, *Eco*RV. B, Southern blot hybridization of *Bam*HI-digested chromosomal DNA of wild type strain (lane 1) and R3KO mutant (lane 2). Left arm for crossover is used as hybridization probe. C, Southern blot hybridization of *Eco*RV and *Bcl*I-digested chromosomal DNA of wild type strain (lane 1 and 2) and R3KO mutant (lane 3 and 4). Deleted part of *sgcR3 *gene is used as hybridization probe. D, Determination of C-1027 production in complementation strains of *sgcR3*. The antibacterial activities against *B. subtilis *of wild type strain (a), R3KO mutant (b), R3KO mutant with pKCR3 (c), R3KO mutant with pSETR3 (d) and R3KO mutant with pLR3 (e) are shown.

In order to ascertain the contribution of *sgcR3 *to the regulation of C-1027 biosynthesis, a part of coding region of *sgcR3 *(507 bp) was replaced with a thiostrepton resistant gene (*tsr*) to create the *sgcR3 *disrupted strain *S. globisporus *R3KO (Fig. 4A). Successful disruption of the intended target was confirmed by PCR using primers complementary to one end of *tsr *and to untouched DNA outside the disruption constructs (data not shown). Southern blot analyses authenticated the site-specific disruptions of *sgcR3 *using left arm for crossover and deleted part of *sgcR3 *gene as probes respectively (Fig. 4B, 4C). The antibacterial bioassay against *B. subtilis *(Fig. 4D, b) and HPLC analysis (Fig. 3d) showed that disruption of *sgcR3 *completely abolished C-1027 production.

To confirm that the disruption of *sgcR3 *was indeed responsible for the abolition of C-1027 production, the mutant was complemented with *sgcR3 *gene. Three *sgcR3 *expression plasmids (pKCR3, pSETR3 and pLR3) were introduced into R3KO mutant by conjugation respectively. pSETR3 and pLR3, both based on the plasmid pSET152 [30] integrating into the ΦC31 *attB *site on the chromosome, had a copy of *sgcR3 *controlled by its native promoter and a strong constitutive promoter ermE*p respectively. The resultant strains with pKCR3 (Fig. 4D, c) and pSETR3 (Fig. 4D, d) restored the C-1027 production and showed dose proportionality as expected. The strain containing pLR3 in which *sgcR3 *was controlled by ermE*p showed less production of C-1027 (Fig. 4D, e) compared with the strain containing pSETR3. No production of C-1027 was detected for the R3KO mutants transformed with pKC1139 and pSET152 (data not shown). These results, fully consistent with those obtained upon overexpression of *sgcR3 *gene, confirmed the positive regulatory role of *sgcR3 *in C-1027 biosynthesis.

### Gene expression analysis in R3KO mutant

**Figure 5:**
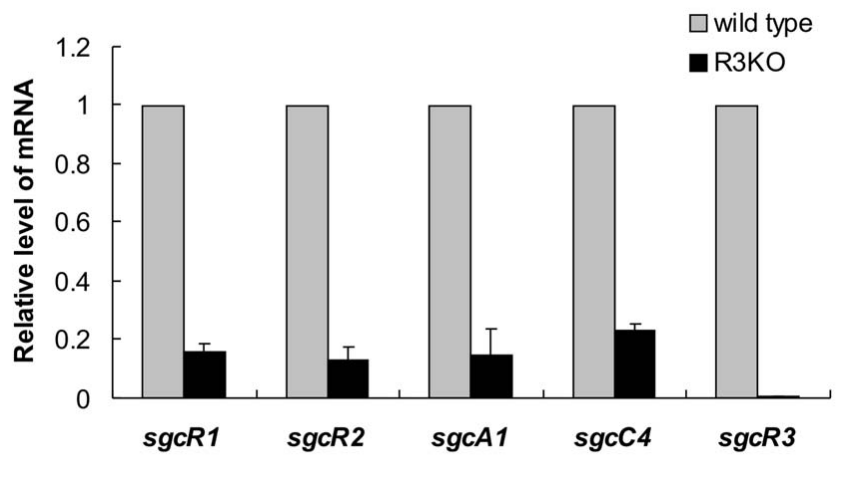
**Transcriptional analyses of different genes in *S. globisporus *C-1027 and R3KO mutant**. The relative abundance of *sgcR1*, *sgcR2*, *sgcA1*, *sgcC4 *and *sgcR3 *transcripts in mycelial patches of wild type strain and R3KO mutant grown on S5 agar plates for 48 h was determined using quantitative real time RT-PCR analysis. Data are from 2 biological samples with 2 determinations each. The values were normalized using values obtained for *hrdB *mRNA and represented as the mean ± SD. The amounts of each particular transcript in wild type strain were expressed as 1.

To investigate the role of *sgcR3 *gene in transcriptional regulation of C-1027 biosynthetic gene cluster, the gene expression analysis was conducted by quantitative real time RT-PCR. The relative level of the transcripts of two other putative regulatory genes, *sgcR1 *and *sgcR2*, and two biochemically characterized structural genes, *sgcA1 *and *sgcC4*, were analysed together with *sgcR3*. The deduced product of *sgcR1 *displays 44% end-to-end identity to StrR, a well-characterized pathway-specific transcriptional activator for streptomycin biosynthesis in *S. griseus *[12]. SgcR2 shares high sequence identity (>40% along the whole length) to AraC/XylS family transcriptional regulators. SgcA1 and SgcC4 were reported to catalyze the first step in the biosynthesis of the deoxy aminosugar and the β-amino acid moieties of C-1027 chromophore respectively [31,32]. Total RNA from the wild type strain and R3KO mutant was extracted under which condition the wild type strain commenced C-1027 production at about 48 h growth on S5 agar. The cDNA was synthesized and then used as template in quantitative PCR. As expected, *sgcR3 *transcripts were almost undetectable in R3KO mutant while readily detectable in wild type strain. Transcripts of the other four genes described above were also readily detected in wild type strain, but were significant lower in the R3KO mutant (13–22% to their counterparts in wild type strain) (Fig. 5). The results indicated that at the onset of the production of C-1027, *sgcR3 *directly or indirectly controlled the expression of not only the structural genes that responsible for the biosynthesis of C-1027 chromophore but also the other two putative regulators situated in the same gene cluster.

### *In trans *complementation of R3KO mutant with *sgcR1R2*

**Figure 6:**
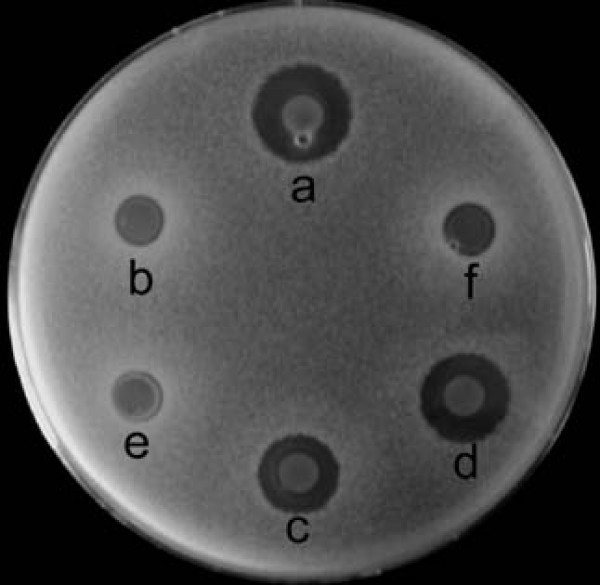
**Determination of C-1027 production in R3KO mutant complemented with *sgcR1R2***. The antibacterial activities against *B. subtilis *of wild type strain (a), R3KO mutant (b), R3KO mutant with pKCR1R2 (c), R3KO mutant with pKCER1R2 (d), R3KO mutant with pKC1139 (e) and R3KO mutant with pSET152 (f) are shown.

The *sgcR1 *and *sgcR2 *were two adjacent genes transcribed in the same direction with a gap of only 25 bp, suggesting that they were transcriptionally coupled within an operon. Confirmation that *sgcR1 *and *sgcR2 *were controlled by *sgcR3 *came from *in trans *complementation of R3KO mutant with *sgcR1R2 *(*sgcR1 *and *sgcR2 *genes). The amplified DNA fragment of *sgcR1R2 *associated with its native promoter was cloned into multi-copy pKC1139 directly or under control of ermE*p to give pKCR1R2 and pKCER1R2. These two plasmids were introduced into *sgcR3 *mutant after conjugal transfer from *Escherichia coli*. C-1027 production was partially restored when *sgcR1R2 *was overexpressed under the control of either the native promoter (Fig. 6c) or ermE*p (Fig. 6d). C-1027 production was not detected in the R3KO mutants in which pKC1139 and pSET152 were introduced (Fig. 6e, 6f). The expression of *sgcR1R2 *functionally complemented the disruption of *sgcR3*, together with results of the gene expression analysis, verified that *sgcR3 *occupied the higher level than *sgcR1R2 *did in the regulatory cascade for C-1027 biosynthesis in *S. globisporus *C-1027.

### Binding of SgcR3 to the *sgcR1R2 *promoter region

**Figure 7:**
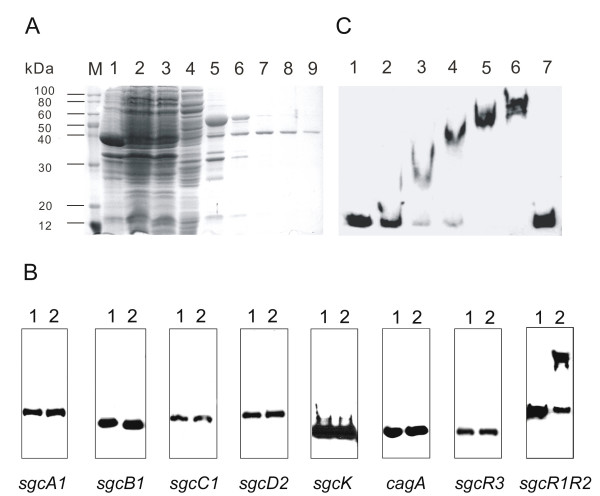
**Gel mobility-shift assays of His_10_-SgcR3 with *sgcR1R2 *promoter region**. A, Purification of recombinant SgcR3 after overexpression as a fusion protein with an N-terminal His_10_-tag in *E. coli *BL21(DE3). Lanes 1, 2, 3 and 4 contain samples of the insoluble lysate of induced cells, the soluble lysate of induced cells, flow through from the column, combined column washes respectively. Lanes 5–9 contain samples of eluates 1–5 eluted by buffer containing 500 mM imidazole. His_10_-SgcR3 protein from the eluate 5 was used in EMSA analysis. The molecular masses (kDa) of the protein markers (TransGen Biotech, Beijing, CN) are indicated. B, EMSA analysis of His_10_-SgcR3 with upstream region of *sgcA1*, *sgcB1*, *sgcC1*, *sgcD2*, *sgcK*, *cagA*, *sgcR3 *and *sgcR1R2*. Each of the lanes contains 20 fmol of fluorescently labeled promoter region DNA fragment. Lanes 2 also contain 13.5 pmol of purified recombinant His_10_-SgcR3 protein. C, EMSA analysis of His_10_-SgcR3 with *sgcR1R2 *promoter region. Each of the lanes contains 20 fmol of fluorescently labeled *sgcR1R2 *promoter region DNA fragment. Lanes 2–6 also contain 0.5 pmol, 3.12 pmol, 6.25 pmol, 13.5 pmol and 27 pmol of purified recombinant His_10_-SgcR3 protein, respectively. Lane 7 contains 6.25 pmol His_10_-SgcR3 and 200 fold excess unlabeled *sgcR1R2 *promoter region DNA fragment.

For further investigation of the function of *sgcR3*, its product was therefore expressed as an N-terminal His_10 _fusion protein in *E. coli *(see Methods). Subsequent SDS-PAGE analysis revealed overproduction of a clone-specific protein of the expected size of His_10_-SgcR3 (45 kDa). This His_10_-tagged SgcR3 protein was purified from the soluble fraction of cell lysate by nickel affinity chromatography and was estimated by SDS-PAGE to be about 90% pure (Fig. 7A, lane 9).

To be a transcriptional activator of C-1027 biosynthesis, SgcR3 was speculated that it may act as a positive regulator by binding at or near the promoter region of biosynthetic genes or regulatory genes and thereby activating their transcription. EMSA were carried out to verify whether SgcR3 indeed had DNA-binding activity, using the purified His_10_-tagged SgcR3 and selected DNA fragments from the biosynthetic gene cluster of C-1027. Eight intergenic regions of interest are chosen for EMSA, including upstream region of *sgcA1*, *sgcB1*, *sgcC1*, *sgcD2*, *sgcK*, *cagA*, *sgcR3 *and *sgcR1R2 *(Fig. 7B). The results showed that the recombinant SgcR3 protein had binding activity to the 455 bp upstream fragment of the *sgcR1R2*, but not for any other of the eight DNA fragments investigated. Further EMSA carried out using different concentration of purified recombinant SgcR3 showed that the shift band emerged along with the increase of the protein amount. Shifting of the labelled probe was not observed when the corresponding unlabelled probes were added in excess to binding reaction (Fig. 7C). Specific binding of SgcR3 to the upstream fragment of the *sgcR1R2 in vitro*, together with the results of gene expression analysis and *sgcR1R2 *cross-complementation in R3KO mutant, indicated that SgcR3 activates the transcription *sgcR1R2 *directly by binding to its promoter region.

## Discussion

The original sequence analysis of the C-1027 biosynthetic gene cluster identified several ORFs whose gene products may have a potential regulatory function [25]. We focused our initial study on the *sgcR3 *gene situated at the right end of the cluster. Overexpression studies with additional copies of *sgcR3 *expressed under the control of its native promoter in wild type strain indicated a positive effect on C-1027 production. The results obtained in the gene disruption experiment clearly demonstrated the essential positive role of *sgcR3 *in regulation of C-1027 biosynthesis.

The results obtained in *sgcR3 *inactivation experiments were proved by complementation of the R3KO mutant using different strategies to express *sgcR3 in trans*. The results showed that expression of *sgcR3 *under the control of its native promoter either introduced by a multi-copy plasmid or integrated into the ΦC31 *attB *site on the chromosome fully restored C-1027 production. Unexpectedly, the complementation of *sgcR3 *under strong constitutive promoter ermE*p produced less C-1027 than under its native promoter, suggesting that the promoter region of *sgcR3 *was intricately regulated for its timing or the amount of expression which was important for the C-1027 production. One possibility is that there is a positive feedback mechanism controlling the expression of *sgcR3*, e.g., SgcR1 and/or SgcR2 can activate the expression of *sgcR3 *in return.

Analysis of gene expression in the mutant and wild type strain suggested that *sgcR3 *control C-1027 production through transcriptional regulation of biosynthetic genes. It also helped to establish a hierarchy among the three regulators of the C-1027 gene cluster. The expression level of *sgcR1 *and *sgcR2 *was significantly lower in R3KO mutant than in wild type strain, implying that *sgcR3 *occupied a higher rung than *sgcR1 *and *sgcR2 *did in the hierarchy of C-1027 regulatory genes. Only TylR among SgcR3 orthologues was characterized by gene disruption, *in vivo *complementation and gene expression experiments [14,23]. Overexpression of TylR was experimentally proved to increase tylosin yield by 60–70% [23]. According to these studies, TylR occupies the lowest level in the genetic hierarchy that controls tylosin production in *S. fradiae*, but that was probably not the case of SgcR3 for C-1027 production in *S. globisporus *C-1027.

Additional evidence for a correlation between these regulators of biosynthesis was observed through the study of cross-complementation experiment. The *sgcR1R2 *functionally complemented R3KO mutant under either its native promoter or strong constitutive promoter ermE*p. Furthermore, the recombinant SgcR3 protein bound specifically to the promoter region of *sgcR1R2*, but not that of *sgcR3 *and some structural genes detected. Therefore, it was very likely that SgcR3 activated the transcription of *sgcR1 *and *sgcR2 *by directly binding to their promoter region, to control the expression of biosynthetic structural genes indirectly. On the other hand, although the recombinant SgcR3 can bind to *sgcR1R2 *promoter region DNA fragment without further macromolecular factor *in vitro*, our results do not completely rule out the possibility that other protein(s) may be required for activating the transcription of *sgcR1R2*.

With few except that no regulatory gene present in the biosynthetic gene cluster, e.g., erythromycin cluster of *Saccharopolyspora erythraea *[33], most much-studied antibiotic gene clusters contain at least one pathway-specific regulator. However, the biosynthesis of more complex molecules may need more regulatory gene products involving a regulatory cascade to affect a positive or negative regulation. Some particularly interesting examples are the tylosin biosynthetic gene cluster of *S. fradiae *[14,18,19,21-23] and the rapamycin biosynthetic gene cluster of *S. hygroscopicus *[16] which contain, remarkably, no fewer than five putative regulatory genes. Further analysis of other ORFs in C-1027 gene cluster revealed that additional three unknown genes might have regulatory role in C-1027 biosynthesis. The *sgcE1 *encodes a protein homologous (43% end-to-end identity) to a transcriptional regulator of HxlR family (GenBank accession no. ABX37987). The *sgcR *encodes a protein demonstrating some homology (20% end-to-end identity) to a transcriptional regulator protein (GenBank accession no. EDS60418) which belongs to XRE (Xenobiotic Response Element) family. The deduced product of *sgcM *was also found to be highly similar to a putative DNA-binding protein of *S. coelicolor *A3(2) with a helix-turn-helix motif (GenBank accession no. NP_630506.1). Both *sgcE1 *and *sgcM *have a highly homologous counterpart in NCS biosynthetic gene cluster of *S. carzinostaticus*. This is not surprising due to the complicated biosynthesis of enediyne chromophore, which involves multiple moieties and a convergent biosynthetic approach used to piece together the final product.

**Figure 8:**
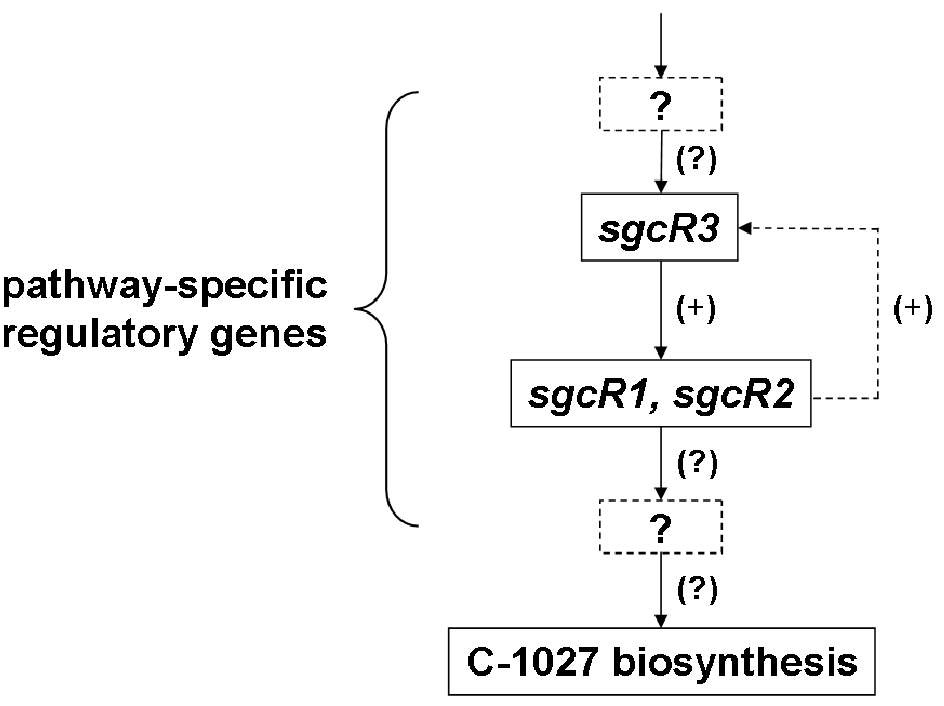
**Hypothetical schematic regulatory hierarchy of C-1027 biosynthesis in *S. globisporus *C-1027**. Break line box with interrogation point represents unknown pathway-specific regulatory genes and break line arrow represents hypothetic feedback regulation. (+) indicates positive regulation and (?) indicates unknown possible regulation.

This work is the first step in deciphering the regulatory factors involved in the biosynthesis of C-1027, and a primary model for pathway-specific regulation of C-1027 production is shown in Fig. 8. Therefore, precise roles for *sgcR3*, *sgcR1*, *sgcR2 *and other putative regulatory genes and their complex interaction remain to be defined. The data presented in this work set the stage for subsequent studies to delineate the complexity of the regulation of C-1027 biosynthesis, as well as for designing strategies for the construction of strains with enhanced C-1027 production.

## Conclusion

The available evidence demonstrated that SgcR3 was a transcriptional activator in C-1027 biosynthesis. Also, *sgcR3 *was demonstrated to occupy a higher level than *sgcR1 *and *sgcR2 *does in the regulatory cascade of C-1027 biosynthesis in *S. globisporus *C-1027 and activate the transcription of *sgcR1R2 *by directly binding to its promoter region.

## Methods

### Strains, media and growth conditions

*E. coli *DH5α was used as host for cloning experiments. *E. coli *ET12567/pUZ8002 [34] was used to transfer DNA into *S. globisporus *by conjugation. *E. coli *BL21 (DE3) (Novagen, Madison, USA) was used to express SgcR3 protein. They were grown either on solid or in liquid Luria-Bertani medium (LB) at 37°C. The following antibiotics were used to select recombinant *E. coli *strains: 100 μg ampicillin (Ap) ml^-1^, 50 μg kanamycin (Km) ml^-1^, 25 μg chloramphenicol (Cm) ml^-1 ^or 50 μg apramycin (Am) ml^-1^. *B. subtilis *CMCC(B) 63501 was used as the test organism for assay of the antibacterial activity of C-1027 [35], grown on solid F403 agar (consisting of 0.5% peptone, 0.3% beef extract, 0.3% K_2_HPO_4 _and 1.5% agar (pH 7.8)) at 37°C.

*S. globisporus *C-1027 (referred to here as wild type strain) and its derivatives were grown at 28°C on S5 agar (consisting of 0.1% KNO_3_, 0.05% NaCl, 0.05% K_2_HPO_4_, 0.001% FeSO_4_, 0.05% MgSO_4_, 2.0% starch and 1.5% agar (pH 7.2)) for sporulation, on mannitol soya flour (MS) agar [34] for conjugation, in the liquid fermentation medium FMC-1027-1 (consisting of 2% dextrin, 0.2% peptone, 1% glycerol, 0.5% corn steep liquor and 0.1% CaCO_3 _(pH 7.0)) for C-1027 production, in Trypticase Soy Broth (TSB, BD, Sparks, USA) for isolation of genomic or plasmid DNA, and maintained as mycelial fragments or spores in 20% (v/v) glycerol at -70°C. The antibiotics apramycin (Am) and thiostrepton (Th) were added at final concentrations of 50 and 30 μg ml^-1 ^to solid medium, and at 10 and 5 μg ml^-1 ^to liquid medium, respectively. Strains used and constructed in this study are listed in Table 1.

**Table 1:** Bacterial strains and plasmids used in this study

Strains or plasmids	Description*	Reference
**Strains**		
*Escherichia coli *DH5α	General cloning host	[36]
*E. coli *ET12567/pUZ8002	Strain used for *E. coli/Streptomyces *conjugation	[34]
*E. coli *BL21 (DE3)	Strain used for the expression of SgcR3 protein	Novagen
*Bacillus subtilis *CMCC(B) 63501	Strain used for C-1027 bioassays	[35]
*Streptomyces globisporus *C-1027	Wild type C-1027 producing strain	[1]
*S. globisporus *R3KO	*S. globisporus *C-1027 with disruption of *sgcR3*, Th^r^	This work
**Plasmids**		
pUC18	General cloning vector, Ap^r^	[36]
pET-16b	*E. coli *expression vector, Ap^r^	Novagen
pKC1139	*E. coli*/*Streptomyces *shuttle vector, Am^r^	[30]
pOJ260	Suicide vector nonreplicating in *Streptomyces*, Am^r^	[30]
pSET152	*Streptomyces *integrative vector, Am^r^	[30]
pL646	pSET152 derivative plasmid containing ermE*p and the ribosome-binding site of the *tuf1 *gene upstream *atrAc *gene, Am^r^	[37]
pOJR3KO	pOJ260 derivative plasmid with the disruption of *sgcR3*, Th^r^	This work
pKCR3	pKC1139 derivative plasmid containing 2,539 bp fragment of *sgcR3 *including its native promoter, Am^r^	This work
pSETR3	pSET152 derivative plasmid containing 2,539 bp fragment of *sgcR3 *including its native promoter, Am^r^	This work
pLR3	pL646 derivative plasmid containing 1,188 bp coding region of *sgcR3 *instead of *atrAc*, Am^r^	This work
pKCR1R2	pKC1139 derivative plasmid containing 2,461 bp fragment of *sgcR1R2 *including its native promoter, Am^r^	This work
pKCER1R2	pKC1139 derivative plasmid containing 2,461 bp fragment of *sgcR1R2 *under the ermE*p, Am^r^	This work

*Abbreviations: Ap^r^, ampicillin resistance; Am^r^, apramycin resistance; Th^r^, thiostrepton resistance.

### DNA manipulations

Standard genetic techniques with *E. coli *and *in vitro *DNA manipulations were as described by Sambrook & Russell [36]. Plasmids used and constructed in this study are listed in Table 1. Recombinant DNA techniques in *Streptomyces *species were performed as described by Kieser et al. [34]. PCR reactions for amplification of indicated DNA products and for verification of gene deletion in *S. globisporus *were carried out using *Pfu *DNA polymerase (TransGen Biotech, Beijing, CN). The primers for PCR amplification are shown in Table 2. Total *S. globisporus *DNA was isolated using the Kirby mix procedure [34]. Southern blot analysis was performed on Hybond-N^+ ^nylon membrane (Amersham Biosciences, Buckinghamshire, UK) with a fluorescein-labelled probe by using the Gene Images Random Prime Labelling Module and CDP-Star Detection Kit (Amersham Biosciences).

**Table 2:** PCR primers used in this study

Name	Sequence	Purpose
WAB1 WAB2	5'-GA**GAATTC**ACCACATGGAACATCTGCTG-3' 5'-GA**AGATCT**GGGAGCACGCAACTGAG-3'	Forward and reverse primers for amplifying 1.4 kbp upstream arm used in knockout of *sgcR3*
		
WCD1 WCD2	5'-A**AGATCT**CTGATCGGCAAGTACGGAGAC-3' 5'-A**AAGCTT**GGTGACAGTTCCGAGGAGGTT-3'	Forward and reverse primers for amplifying 1.4 kbp downstream arm used in knockout of *sgcR3*
		
H1 H2	5'-CA**GAATTC**TCTCCTACGCCCTGCTCATC-3' 5'-AC**TCTAGA**AGAACCGCTCGGCCTGTC-3'	Forward and reverse primers for amplifying 2,539 bp *sgcR3 *including its native promoter
		
sgcR3_FOR sgcR3_REV	5'-G**CATATG**GCGGCACACGACGTGAG-3' 5'-A**GGATCC**CAGCTCCCCTCCTGGGAG-3'	Forward and reverse primers for amplifying 1,188 bp *sgcR3 *coding region
		
P17 P18	5'-AT**GGATCC**GCCCCTCGCTCATTGTCG-3' 5'-AT**GAATTC **CGGATCGGATGTGCTGGTCT-3'	Forward and reverse primers for amplifying 2,461 bp *sgcR1R2 *including its native promoter
		
hrdBRT1 hrdBRT2	5'-TGGTCGAGGTCATCAACAAG-3' 5'-TGGACCTCGATGACCTTCTC-3'	Forward and reverse primers used for the detection of *hrdB *transcripts
		
R1RT1 R1RT2	5'-GAAAAGTGACTCTGCCCAACGC-3' 5'-CGCTGCACATGGGAACGATC-3'	Forward and reverse primers used for the detection of *sgcR1 *transcripts
		
R2RT1 R2RT2	5'-ACCACGAACACCATCGAGGAC-3' 5'-CGGAAGATGCGGGTGAAATG-3'	Forward and reverse primers used for the detection of *sgcR2 *transcripts
		
R3RT1 R3RT2	5'-GTGGGCGAACGGGAGACGGT-3' 5'-TGCCGAGGGCCGATCAGAGG-3'	Forward and reverse primers used for the detection of *sgcR3 *transcripts
		
A1RT1 A1RT2	5'-CAGAACATATTCAAGGACTGGC-3' 5'-CACTTGTACTGTCGGGTGGTT-3'	Forward and reverse primers used for the detection of *sgcA1 *transcripts
		
C4RT1 C4RT2	5'-CTGTGGCTACGGACGAGATTG-3' 5'-CCGAACAGAACATCCCCATCT-3'	Forward and reverse primers used for the detection of *sgcC4 *transcripts
		
sgcA1p_FOR_F sgcA1p_REV	5'-CAGCTGGAGTACGCGATGG-3' 5'-CGACAATGGCGACCTC TTTG-3'	Forward and reverse primers for amplifying 438 bp upstream of *sgcA1*
		
sgcB1p_FOR_F sgcB1p_REV	5'-GAGGGGTTTTGAGGGGTGAA-3' 5'-GATAGGGGTCGGGGGACATC-3'	Forward and reverse primers for amplifying 496 bp upstream of *sgcB1*
		
sgcC1p_FOR_F sgcC1p_REV	5'-GGTGCAACACCGCAGATCC-3' 5'-GAGCTGCGCGGAAGGAAG-3'	Forward and reverse primers for amplifying 481 bp upstream of *sgcC1*
		
sgcD2p_FOR_F sgcD2p_REV	5'-CCTGGAACCATCCGCGAAAC-3' 5'-CGGTCGATCACCAGCACTTC-3'	Forward and reverse primers for amplifying 448 bp upstream of *sgcD2*
		
sgcKp_FOR_F sgcKp_REV	5'-AGGATCATCGTCCCGTCTC-3' 5'-GAGCCAGTCGAACGACTCCA-3'	Forward and reverse primers for amplifying 471 bp upstream of *sgcK*
		
R1R2p_FOR_F R1R2p_REV	5'-ACGATCGTGTCCGCGTAGG-3' 5'-AACGATCGATTCCGTTCAGG-3'	Forward and reverse primers for amplifying 455 bp upstream of *sgcR1R2*
		
R3p_FOR_F R3p_REV	5'-GCCCACGCCCTCTGATCG-3' 5'-CGGGACCATGCTCCGTGTA-3'	Forward and reverse primers for amplifying 467 bp upstream of *sgcR3*
		
cagApEMSA1 cagApEMSA2	5'-CACGCTCTGTCCGTCACTCA-3' 5'-ATCCGCTCCCGAAGGTGG-3'	Forward and reverse primers for amplifying 429 bp upstream of *cagA*

Restriction endonuclease recognition sequences introduced by these oligonucleotides are bold.

### Construction of expression plasmids

Three plasmids for *sgcR3 *expression were constructed as follows. The *sgcR3 *with its promoter region (2,539 bp) was amplified by PCR and then cloned into the *E. coli*/*Streptomyces *shuttle vector pKC1139 [30] to give pKCR3. The fragment was also ligated into an integrative vector pSET152 [30] to give pSETR3. The *sgcR3 *coding region (1,188 bp) amplified by PCR was introduced to pL646 [37], displacing *atrAc *gene under the control of a strong constitutive promoter ermE*p, to give pLR3.

Similarly, *sgcR1R2 *(2,461 bp) with its promoter region were amplified by PCR and cloned into pKC1139 vector to yield pKCR1R2. This fragment was also cloned into pKC1139 under the control of ermE*p, resulting in plasmid pKCER1R2.

### Disruption of *sgcR3*

The disruption construct consists of a thiostrepton resistant gene (*tsr*), sandwiched between two PCR products ("arms") that each contains sequence from *sgcR3 *plus flanking DNA. The arms (which were authenticated by sequence analysis) were of approximately equal size (1.4 kbp).

The primers for *sgcR3 *disruption introduced restriction sites into the arms (*Eco*RI and *Bgl*II in the upstream arm, *Bgl*II and *Hin*dIII in the downstream arm), and thus allowed fusion at the *Bgl*II sites by ligation into pUC18. Then, the *tsr *fragment (a 1 kbp *Bcl*I restriction fragment from pIJ680 [34]) was introduced into the *Bgl*II site and thereby displaced 507 bp of *sgcR3*. Disrupted *sgcR3 *plus flanking DNA (approximate 3.8 kbp in total) was ligated into suicide plasmid pOJ260 [30] to give pOJR3KO. This plasmid was introduced by transformation into *E. coli *ET12567/pUZ8002 and then transferred into *S. globisporus *C-1027 by conjugation. Double-crossover exconjugants were selected on MS agar containing Th and Am (Th^r^, Am^s^). Deletions within *sgcR3 *were confirmed by PCR and Southern blot hybridization.

### Gene expression analysis by real time reverse transcriptase PCR (RT-PCR)

RNA was isolated from *S. globisporus *mycelia scraped from cellophane laid on the surface of S5 agar plates, treated with DNaseI (Promega, WI, USA) and quantitated as described previously [37,38]. The first strand synthesis of cDNA was performed with SuperScript III First-strand Synthesis System (Inivitrogen, CA, USA) using 2 μg total RNA and the random hexamers as primers following the manufacturer's instructions. Oligonucleotides were designed to amplify fragments of about 100–150 bp from the target genes (Table 2).

Quantitative real time PCR of selected genes was performed using the SYBR Green PCR Master Mix (ABI, Cheshire, UK). To control for genomic DNA contamination, each sample was also incubated with a reaction mixture that lacked RT. Real time PCR conditions were as follows: 94°C for 10 min, 40 cycles of 94°C for 30 s, 60°C for 30 s and 72°C for 30 s. A step of 78°C for 10 s during which fluorescence was measured was included at the end of each cycle. The reactions were subjected to a heat dissociation protocol after the final cycle of PCR to indicate the proper temperature for fluorescence detection. After PCR amplifications, data were analyzed with iQ5 Optical System version 1.1.1442.0 Software (Bio-Rad). The threshold cycle (Ct) was calculated from the programme. Serial dilution of the cDNA was subjected to real time PCR. For each transcript, plots of Log_2_(dilution factor) against the Ct values provided an estimate of the efficiency of the amplification. The target gene mRNA level were normalised internally to the level of *hrdB *mRNA according to the Pfaffl's method [39].

### C-1027 production and analysis

For C-1027 production, *S. globisporus *strains were grown in liquid medium FMC-1027-1 by a two-stage fermentation. The spore suspensions of the different strains were adjusted to the same concentration for inoculation. The seed inoculum was prepared by inoculating 100 ml of FMC-1027-1 with an aliquot of the spore suspension and incubating the mixture at 28°C and 250 rpm for 2 days. The seed culture (5%) was added to a fresh 100 ml of FMC-1027-1, continuing to incubate at 28°C and 250 rpm for 5 days. To obtain statistically significant results, each strain was represented by a triplicate sample set. Dry weight of mycelia was measured in cultures taken at different time points in the fermentation course and the pattern of growth curves were monitored consistently among the relevant strains. The production of C-1027 was analyzed using the fermentation supernatants of relevant strains with the same growth curves.

C-1027 production was detected by assaying its antibacterial activity against *B. subtilis *[35]. The fermentation supernatant (180 μl) was added to stainless steel cylinders placed on F403 agar plate containing *B. subtilis *spores (0.4% v/v). C-1027 production was estimated by measuring the sizes of the inhibition zones after incubated at 37°C for 12 h.

Isolation and high-pressure liquid chromatography (HPLC) analysis of C-1027 chromophore were carried out mostly following Liu et al. [25]. Briefly, (NH_4_)_2_SO_4 _was added to the 250 ml fermentation supernatant of relevant strains to 100% saturation and then adjusted to pH 4.0 with 0.1 M HCl. The precipitated C-1027 chromoprotein was dissolved in 15 ml 0.1 M potassium phosphate (pH 8.0). The supernatant was then extracted with 50 ml ethyl acetate (EtOAc), concentrated in vacuum, and re-dissolved in 250 μl methanol. 25 μl cleared sample was subjected to HPLC on a Kromasil C-18 column (5 μm, 150 × 4.6 mm, Bohus, SE), eluted isocratically with 20 mM potassium phosphate (pH 6.86)/CH_3_CN (50:50, v/v) at a flow rate of 1.0 ml/min and detected by monitoring UV absorbance at 350 nm. The C-1027 enediyne chromophore standard for HPLC analysis was confirmed by ESI-MS.

### Expression and purification of His_10_-tagged SgcR3

The *sgcR3 *coding sequence was PCR-amplified from *S. globisporus *C-1027 genome DNA containing an *Nde*I and *Bam*HI restriction sites, and then ligated into pET-16b (Novagen, Madison, USA), authenticated by sequencing, and then transformed into the *E. coli *BL21 (DE3). For production of His_10_-tagged SgcR3, cultures (800 ml; OD_600 _= 0.6) were induced with IPTG (0.05 mM final), incubated at 28°C for 6 h, harvested by centrifugation. The cell suspension was sonicated for 60 × 10 s with 10 s intervals between each treatment in 30 ml lysis buffer (50 mM NaH_2_PO_4_, pH 8.0, 300 mM NaCl, 10 mM imidazole, 2 mg lysozyme ml^-1^). Cellular debris was removed by centrifugation (12,000 rpm for 10 min). His_10_-tagged SgcR3 was then affinity purified using HisTrap™ FF crude (Amersham Biosciences) according to the manufacturer's directions and fractions eluted from the column were analysed on SDS-12% w/v polyacrylamide gels. Those fractions containing recombinant protein were pooled, dialysed overnight at 4°C against dialysis buffer (25 mM Tris/HCl (pH 7.5), 10% (w/v) glycerol, 2 mM DTT) and stored at -70°C. The BCA™ Protein Assay Kit (Pierce Biotechnology, Rockfold, USA) was used for protein quantification with bovine serum albumin as the standard.

### Electrophoretic mobility shift analysis (EMSA)

DNA fragments upstream of *sgcR1R2*, *sgcR3*, *sgcA1*, *sgcB1*, *sgcC1*, *sgcD2*, *sgcK *and *cagA *were generated by PCR using *S. globisporus *C-1027 genomic DNA as template. Primers are shown in Table 2. After purification by agarose electrophoresis, these DNA fragments were 3'-end labelled with Biotin-11-ddUTP using the Biotin 3' End DNA Labeling Kit (Pierce Biotechnology). Probes were incubated at 4°C for 20 min with purified His_10_-SgcR3 protein in binding buffer (100 mM Tris/HCl (pH 7.5), 500 mM KCl, 10 mM DTT). Reaction mixtures were then analysed by non-denaturing PAGE (5% w/v gels) in 0.5 × TBE buffer at 4°C. The gel was then transferred to nylon membrane (Amersham Biosciences) by electrophoretic transfer. The biotin end-labeled DNA was detected by LightShift Chemiluminescent EMSA Kit (Pierce Biotechnology) according to the manufacturer's instructions.

## Authors' contributions

LW carried out the main experimentation and drafted the manuscript. YH and YZ constructed some of the plasmids, SW and ZC participated in fermentation of *S. globisporus*, YB participated in statistical analysis of the real time RT-PCR, WJ participated in the HPLC experiments. BH conceived, designed and coordinated the study and revised the manuscript. All authors have read and approved of the final manuscript.
